# The mitochondrial inhibitor oligomycin induces an inflammatory response in the rat knee joint

**DOI:** 10.1186/s12891-017-1621-2

**Published:** 2017-06-12

**Authors:** Carlos Vaamonde-García, Jesús Loureiro, Marta N. Valcárcel-Ares, Romina R. Riveiro-Naveira, Olalla Ramil-Gómez, Laura Hermida-Carballo, Alberto Centeno, Rosa Meijide-Failde, Francisco J. Blanco, María J. López-Armada

**Affiliations:** 1 Aging and Inflammation Research Lab, Instituto de Investigación Biomédica de A Coruña (INIBIC), Complexo Hospitalario Universitario A Coruña (CHUAC), Sergas, Universidade da Coruña (UDC), As Xubias, 15006 A Coruña, Spain; 2 Tissue Engineering and Cellular Therapy Group, INIBIC, Department of Medicine, Faculty of Health Sciences- UDC, Campus Oza, A Coruña, Spain; 3 Experimental Surgery Unit, INIBIC-CHUAC, A Coruña, Spain; 4 Osteoarticular and Aging Research Lab, Rheumatology Service, INIBIC, CHUAC, Sergas, UDC, A Coruña, Spain

**Keywords:** Mitochondria, Inflammation, Oxidative stress, Synovial tissue, Cartilage

## Abstract

**Background:**

Recent findings support a connection between mitochondrial dysfunction and activation of inflammatory pathways in articular cells. This study investigates in vivo in an acute model whether intra-articular administration of oligomycin, an inhibitor of mitochondrial function, induces an oxidative and inflammatory response in rat knee joints.

**Methods:**

Oligomycin was injected into the rat left knee joint on days 0, 2, and 5 before joint tissues were obtained on day 6. The right knee joint served as control. Results were evaluated by macroscopy and histopathology and by measuring cellular and mitochondrial reactive oxygen species (ROS), 4-hydroxy-2-nonenal (4-HNE, a marker of lipid peroxidation), nuclear factor erythroid 2-related factor 2 (Nrf2), and CD68 (macrophages) and chemokine levels. The marker of mitochondrial mass COX-IV was also evaluated.

**Results:**

The macroscopic findings showed significantly greater swelling in oligomycin-injected knees than in control knees. Likewise, the histological score of synovial damage was also increased significantly. Immunohistochemical studies showed high expression of IL-8, coinciding with a marked infiltration of polymorphonuclears and CD68+ cells in the synovium. Mitochondrial mass was increased in the synovium of oligomycin-injected joints, as well as cellular and mitochondrial ROS production, and 4-HNE. Relatedly, expression of the oxidative stress-related transcription factor Nrf2 was also increased. As expected, no histological differences were observed in the cartilage; however, cytokine-induced neutrophil chemoattractant-1 mRNA and protein expression were up-regulated in this tissue.

**Conclusions:**

Mitochondrial failure in the joint is able to reproduce the oxidative and inflammatory status observed in arthritic joints.

**Electronic supplementary material:**

The online version of this article (doi:10.1186/s12891-017-1621-2) contains supplementary material, which is available to authorized users.

## Background

Multiple studies indicate that reactive oxygen species (ROS) production is increased in rheumatic diseases (e.g., rheumatoid arthritis (RA) or osteoarthritis (OA)) leading to oxidative stress which may contribute to articular damage and hence to the chronicity of these pathologies. In fact, a correlation between the disease activity of arthritis and the presence of oxidative stress has been described [1, 2]. ROS and oxidative damage including lipid peroxidation, protein oxidation, or DNA lesions actively participate in pathological processes in the joint, among them, actively participate in pathological processes in the joint, including activation of inflammatory and destructive responses [1, 3]. Thus, the over-activation of pro-inflammatory pathways in the joint drives the pathogenesis of articular diseases [4–6]. Likewise, synovial inflammation is strongly correlated with progression of inflammatory arthritis [7], and similarly, plays an important role in the onset of articular diseases not classically considered inflammatory such as OA [4, 6]. Finally, drugs commonly used in treatment of chronic arthropathies exert their effect through modulating oxidative processes [1, 8].

Mitochondria play a pivotal role in maintaining of redox homeostasis since, they are both the major producers and the major targets of ROS in the cell [9]. As a consequence, oxidative damage to mitochondria may lead to respiratory chain dysfunction, which results in increased ROS levels, leading to a vicious cycle of oxidative stress [10]. In fact, a decline in mitochondrial function has been linked to rheumatoid disorders [11–16]. Specifically, a deficiency in one subunit of the mitochondrial respiratory chain (MRC) complex IV is observed in the synovium of RA and juvenile idiopathic arthritis (JIA) [17, 18]. Moreover, OA chondrocytes show decreased activity of MRC complexes I, II, and III [13]. Additionally, a growing number of studies have demonstrated the involvement of mitochondrial damage in different aspects of articular diseases, e.g., autoimmunity [19], or the hypoxic state of synovial tissue in RA [17], and several pathways associated with cartilage degradation in OA pathology [16, 20–25]. Pro-inflammatory mediators, which are highly produced in these disorders, may also alter mitochondrial function of human synoviocytes and chondrocytes [22, 26–28], reinforcing the strong link between mitochondrial defects and oxidative stress and inflammation [29].

Previously, it has been shown that oligomycin, an inhibitor of mitochondrial complex V, induces an oxidative, inflammatory, and destructive response in cultured human normal synoviocytes and chondrocytes [20, 23, 30]. However, despite the potential involvement of mitochondrial dysfunction in arthropathies, no studies in vivo have evaluated the direct effect of mitochondrial damage by oligomycin on the joint. Therefore, the aim of this study was to analyze in vivo in an acute model whether mitochondrial defects can reproduce the oxidative and inflammatory status observed in arthritic joints. Additionally, we examined whether a differential response could take place in articular tissues. Finally, we also evaluated the expression of the nuclear factor erythroid 2-related factor 2 (Nrf2), a redox-sensitive transcription factor responsible for preserving the cellular defense against oxidative overload [31, 32].

## Methods

### In vivo model of joint damage induced by mitochondrial dysfunction

Twenty-seven female *Wistar* rats (Harlan Interfauna Ibérica, Barcelona, Spain) weighing between 180 and 220 g (4 months) were used. The animals were kept at room temperature (20-24 °C) and commercial food and water was available ad libitum. Rats were randomly assigned into three groups: healthy group (in which rats were not injected); oligomycin group (O4876, Sigma, San Luis, MO, USA) employed as mitochondrial complex V inhibitor [30, 33, 34] (in which the left knee joints were injected intra-articularly with 20 μg oligomycin in 30 μl vehicle [oligomycin-injected joints], and the right knee joints were injected with an equal volume of vehicle [oligomycin-vehicle], containing the same amount of DMSO); and the lipopolysaccharide (LPS) group (Sigma), employed as positive control of inflammatory signs [35, 36] (in which the left knee joints were injected intra-articularly with 10 μg LPS in 30 μl vehicle [LPS-injected joints], and the contralateral joints with its vehicle [LPS-vehicle]). The dose of oligomycin was chosen based on prior works with this mitochondrial inhibitor in rats [34] and pilot experiments where we found that the employed dose, 0.1 mg/kg, had an inflammatory response and was neither signs of acute toxicity (convulsion, hypoactivity, weakness and ataxia). The oligomycin was dissolved in Dimethyl Sulfoxide (DMSO) at 50 mg/ml and stored at −20 °C. For injection, the stock solution was mixed with 0.1% bovine serum albumin (BSA) in Phosphate buffered saline (PBS). All animal manipulations and intra-articular injections, which were carried out by a 26G needle, were performed under Sevorane™ (AbbVie, Madrid, Spain) anesthesia. The time course of this acute articular model is based on previous literature [35, 37]. Intra-articular injections were carried out on days 0, 2, and 5, and rats were euthanized on day 6 being deeply anesthetized with Sevorane™ and after performing the extraction of the blood, their deaths will be induced by anesthetic overdose. Hind joints were dissected and processed either as a whole, or joint tissues were obtained separately. Five animals for each group (healthy, oligomycin and LPS) were employed to make the histological analysis and immunohistochemistry studies; 7 other animals for oligomycin group were employed to evaluate, in synovial membrane, the cytosolic and mitochondrial ROS, cytochrome c oxidase subunit IV (COX-IV), and CINC-1 levels by ELISA in cartilage; and 5 more animals for oligomycin group were used to analyze the cartilage gene expression.

### Human samples

Human synovial tissue was obtained at time of total joint replacement surgery or above-the-knee amputations from patients with no history of joint disease (mean ± SD age 74 ± 8 years; *n* = 8), or with OA (mean ± SD age 70 ± 7 years; *n* = 8). All studies were performed strictly in accordance with current local ethics regulations.

### Joint swelling score

The severity of arthritis, indicated by joint swelling, was quantified by measuring the knee joint diameter using a digital caliper (S-CalWork, Sylvac, Malleray, Switzerland) by two blinded observers on days 0, 2, 5, and 6. The results are expressed as the difference (delta, Δ) between the joint size in mm at days 2, 5, or 6 and the joint size before the first intra-articular injection (day 0).

### Histological analysis

Knee joints from rats injected intra-articularly with oligomycin and its vehicle (*n* = 5) were dissected, fixed in 4% formaldehyde in PBS, decalcified with Shandon TBD-1 (Thermo Fisher Scientific Inc., Waltham, MA, USA), and embedded in paraffin (frontal section). Lesions in the synovial tissue and cartilage were evaluated by semi-quantitative analysis by two blinded researchers using an Olympus microscope (Olympus BX61, Olympus Biosystems, Barcelona, Spain). Sections (4-μm thick) of the joint were stained with hematoxylin and eosin or Safranin O-fast green (Merck, Madrid, Spain) for this purpose. Two samples per animal and five microscopic fields each sample at 20X magnification were used to evaluated damage score for synovial and cartilage tissue, respectively. According to the semi-quantitative modified OARSI score, the grade of the synovial lesion was scored from 0 to 4 (0 being not damaged and 4 most damaged), and the evaluation parameters were: numbers of lining cell layers, proliferation of the subintima tissue, infiltration of inflammatory cells, and pannus formation [38]. The grade of the cartilage lesion was scored from 0 to 13 (0 being not damaged and 13 most damaged) by evaluating the structure, cellular abnormalities, and matrix staining of the cartilage [39]. The percentage of polymorphonuclear leukocytes (PMN) in the synovial infiltrate was identified by evaluating hematoxylin and eosin-stained joint sections.

### Immunohistochemistry and immunofluorescence

IL-8, CD-68, Nrf2, and 4-hidroxi-2-nonenal (4-HNE) were evaluated on sections rats from paraffin-embedded joint, while those for COX-IV were evaluated on frozen sections. Additionally, Nrf2 immunohistochemistry analysis was also carried out on synovial tissue from healthy and OA patients. Paraffin-embedded sections were first deparaffinized with xylene and then rehydrated in graded ethanol and water. For antigen unmasking, sections were placed in citrate buffer (pH 6.0; Dako, Glostrup, Denmark) and heated in a pressure cooker. Endogenous peroxidase activity was quenched using a commercial reactive (Dako) for 10 min. After washing in PBS, slides were incubated overnight at 4 °C with anti-IL-8 (1:150, Abcam, Cambridge, UK) or anti-CD68 (1:100, Abcam) antibodies, or 1 h at room temperature with anti-Nrf2 (1:300; Abcam) or anti-4-HNE (1:150, Abcam) antibodies. Bound antibodies were detected with a secondary antibody and diaminobenzidine using the commercial EnVision™ Detection System (Dako). Finally, sections were counterstained with Gill III hematoxylin (Merck) and mounted with DePeX (Sigma). For COX-IV analysis, frozen cryostat sections (6-μm thick) of synovial membrane were fixed for 15 min in 4% formaldehyde in PBS, and blocked with 10% horse serum for 1 h in PBS with 0.3% Triton X-100. A rabbit anti-COX-IV (1:200; Abcam) antibody was incubated in PBS with 0.1% Triton X-100 overnight at 4 °C. After rinse, secondary antibody, donkey anti-rabbit antibody alexa 488 (1:500; BD Biosciences Pharmigen, San Diego, CA, USA; fluorescence signal green) was incubated during 1 h at room temperature. Another washing process was then applied, and finally the slides were counterstained with the nuclear marker 4′,6-diamidino-2-phenylindole (DAPI, Sigma; fluorescence signal blue), and coverslipped using fluorescent mounting medium (Dako). Multiple images of each slide were captured with a computer-controlled digital camera (Olympus BX61, Olympus). By using an image processing software (Image J software, http://imagej.nih.gov/), the area of the synovial tissue presenting a positive signal of immunostaining was measured and represented as the percentage of the positive area of synovial tissue in relation to total area of synovium*.*

### In situ detection of cytosolic and mitochondrial ROS

ROS levels in the synovial tissue from knee joints injected intra-articularly with oligomycin or its vehicle were analyzed ex vivo by a modified method for measurement of ROS generation using superoxide-sensitive fluorophores [40]. The oxidative fluorescent indicator dihydroethidium (DHE; Sigma; fluorescence signal red) was used to evaluate the cytosolic production of ROS, whereas the mitochondrial one was estimated using the MitoSOX™ Red Mitochondrial Superoxide Indicator (MitoSOX, Life Technologies, Carlsbad, CA, USA; fluorescence signal red). Frozen sections of synovial membrane (6 μm) were incubated with DHE (2 μM) or MitoSOX (1 μM) in a humidified chamber and protected from light for 30 min at 37 °C. Then, sections were fixed for 15 min in 4% paraformaldehyde in PBS, counterstained with the nuclear marker DAPI (fluorescence signal blue), and coverslipped using fluorescent mounting medium. To detect red fluorescence, excitation was at 405 nm with the emission collected through a 605/15 filter. To validate the specificity of the fluorescence probes, adjacent slides were preincubated with the superoxide dismutase (SOD; 250 U/ml; Sigma) for 30 min at 37 °C before coincubation with ROS detectors. Multiple images were captured with a computer-controlled digital camera (Olympus BX61, Olympus) and the positive DHE or MitoSOX fluorescence signal (red) of whole synovial tissue was quantified by Image J software and normalized with DAPI (blue).

### Analysis of gene expression

Cartilage slices were obtained from the rat knee joint, and samples were frozen, and powdered. RNA from tissue was extracted with the TRIzol Reagent method (Invitrogen, Paisley, UK), and RNA (1 μg) was treated with DNase (Invitrogen), and retro-transcribed with Superscript® VILO™ (Invitrogen). Real-time polymerase chain reaction analysis for gene expression of cytokine-induced neutrophil chemoattractant-1 (*CINC-1*) and of the housekeeping gene hypoxanthine phosphoribosyltransferase (*HPRT*) was performed using a LightCycler 480 SYBR Green I Master kit and the LightCycler 480 II PCR system (Roche Diagnostics, Abingdon, UK) by delta delta CT analysis method. The gene-specific primer pairs used were as follows: *CINC-1*, forward 5′-cacactccaacagagcacca-3′, reverse 5′-tgacagcgcagctcattg-3′; and *HPRT*, forward 5′-gaccggttctgtcatgtcg-3′, reverse 5′-acctggttcatcatcactaatcac-3′.

### CINC-1 analysis by ELISA

Cartilage slices from knee joints injected intra-articularly with oligomycin or its vehicle were obtained upon sacrifice and incubated 24 h at 37 °C in Dulbecco’s modified Eagle’s medium (DMEM, Gibco Life Technologies, Paisley, UK). Rat CINC-1 content in the supernatants was measured with an ELISA (Quantikine® R&D System, Abingdon, UK) according to the manufacturer’s recommendations. In order to normalize the obtained concentrations, total DNA from tissue was extracted by TRIzol Reagent method. Data are expressed as pg protein released per μg of DNA.

### Statistical analysis

Data are presented as the mean ± SEM or as representative results, as indicated. Tissues from different animals were pooled when indicated. The GraphPad PRISM version 5 statistical software package (La Jolla, CA, USA) was used to compare experimental groups by Wilcoxon’s paired comparison test. In addition, we employed the Mann-Whitney U test to analyze the differences in Nrf-2 expression from synovial tissue of healthy vs. OA patients. *P* < 0.05 was considered statistically significant.

## Results

### Oligomycin induces joint swelling and inflammatory changes in the synovial tissue

**Fig. 1:**
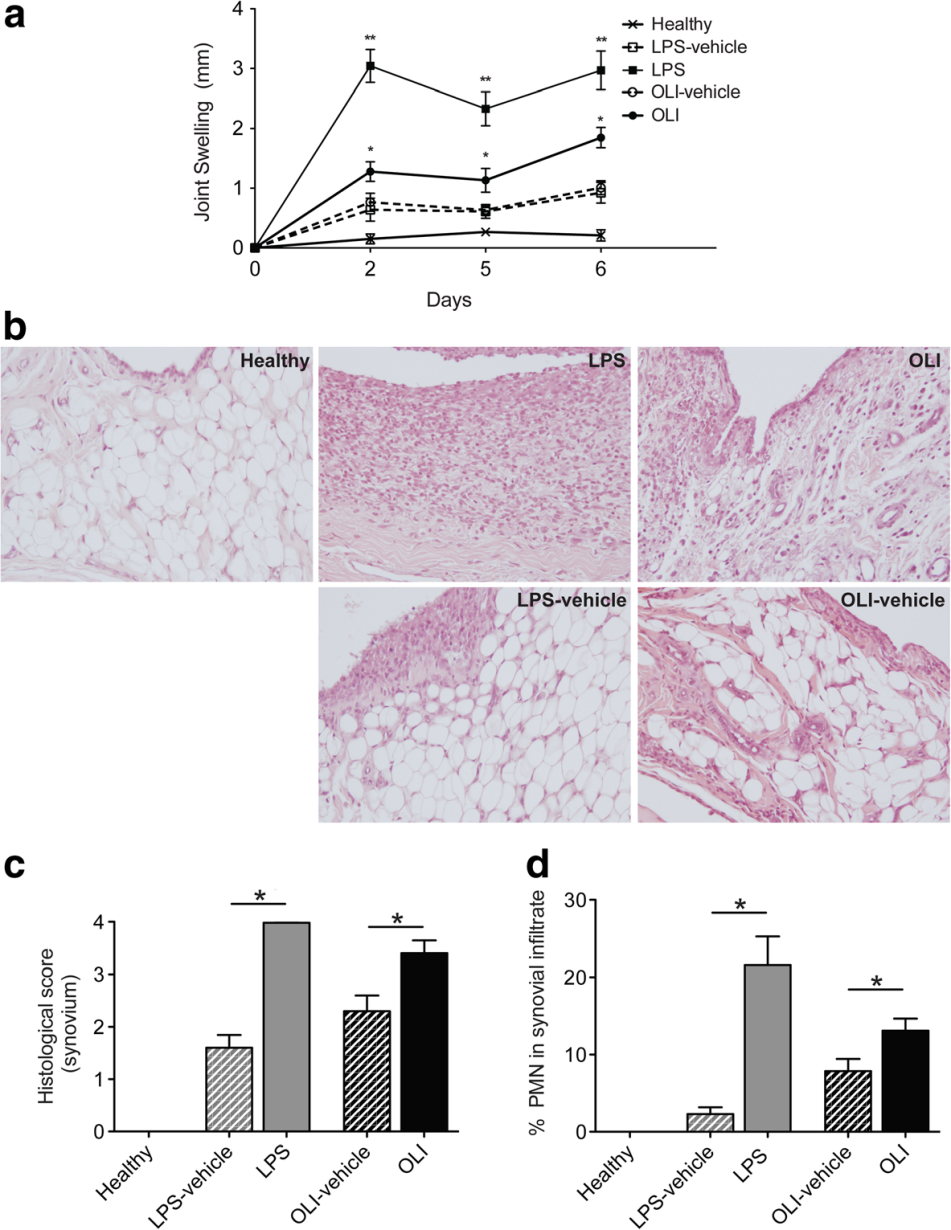
Oligomycin can induce joint swelling and inflammatory changes in the synovial tissue. **a** Knee joint swelling as an indicator of inflammation is estimated from joint width (as described in Methods). Values are mean ± SEM (*n* = 5). *P ≤ 0.005 and **P ≤ 0.001 vs contralateral vehicle-injected knees. **b** Representative images of synovial tissue stained with hematoxylin and eosin from each group of study. **c** Semi-quantitative score of pathological alterations in synovial tissue as described in Methods. Data represent mean ± SEM (*n* = 5 independent samples for each condition). *P ≤ 0.05 vs contralateral vehicle-injected knees. **d** Percentage of PMN was analyzed as described in Methods. Values are mean ± SEM (*n* = 5 independent synovial tissues for each condition). *P ≤ 0.05 vs contralateral vehicle-injected knees. LPS, lipopolysaccharide; OLI, oligomycin

Animals injected with oligomycin developed significantly more joint swelling than contralateral oligomycin-vehicle knees (*P* ≤ 0.005) (Fig. 1a). Oligomycin clearly increased articular swelling by day 2, and maintained incremental increases in swelling over time, achieving the highest changes on day 6 (oligomycin: 1.9 ± 0.2 mm, oligomycin-vehicle: 1.0 ± 0.1 mm). As expected, LPS-injected knees also significantly increased in joint thickness (*P* ≤ 0.001).

**Fig. 2:**
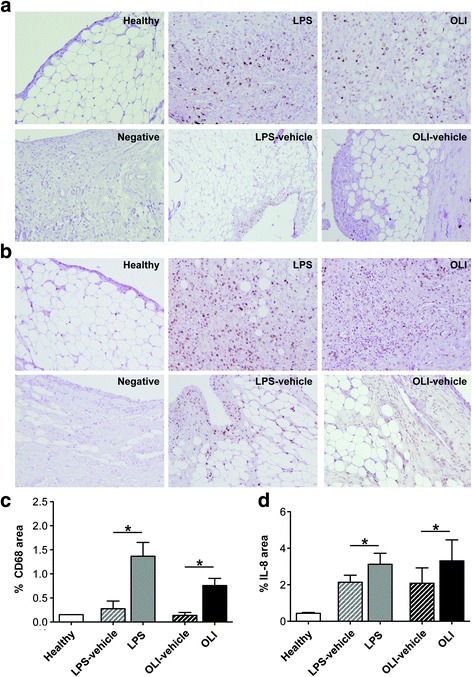
Mitochondrial dysfunction increases CD68 (panel **a**) positive cells and IL-8 (panel **b**) in the synovial membrane. **a** Representative samples of CD68 immunohistochemistry in synovial membrane. **b** Synovium sections were analyzed by immunohistochemistry for IL-8. Quantitative analysis of CD68 (**c**) and IL-8 (**d**) positive cells. Values represent mean ± SEM (*n* = 5 independent synovial tissues). To assess non-immune non-specific binding negative control was included in each experiment. * *P* ≤ 0.05 vs contralateral vehicle-injected knees. LPS, lipopolysaccharide; OLI, oligomycin

Pathological alterations in synovial tissue were analyzed by conventional staining methods. Oligomycin-injected joints showed evident synovitis, with a clear increase in the number of lining cell layers, proliferation of subintima tissue, and cell infiltration (Fig. 1b). In agreement with the results of previous studies [35, 36], LPS-injected joints exhibited significant synovial hyperplasia, with a high number of inflammatory cells (Fig. 1b). The semi-quantitative modified OARSI score confirmed significantly more severe pathological changes observed in the synovium of oligomycin-injected joints than of vehicle-injected control knees (*P* ≤ 0.05) (Fig. 1c). In particular, morphological analysis showed that after oligomycin injection, there was substantial infiltration of PMN into the synovial membrane (Fig. 1d). See Additional file 1: Figure S1 for complete histology images. Moreover, we also confirmed, by immunohistochemical analysis, a significantly higher number of CD68+ cells in the synovial infiltrate from LPS- and oligomycin-injected joints than from vehicle-injected control joints (Fig. 2a and c) (*P* ≤ 0.05).

IL-8 is a chemokine characterized by attracting neutrophils at the site of inflammation, which plays a pivotal role in arthritis pathogenesis [5]. By immunohistochemical analysis, we evaluated the IL-8 protein expression in the synovium. The results indicated significantly (*P* ≤ 0.05) higher IL-8 protein expression in the synovial tissue of LPS- and oligomycin-injected joints than of vehicle-injected control knees (Fig. 2b and d).

### Oligomycin triggers an oxidative response and a mitochondrial alteration in the synovial tissue

**Fig. 3:**
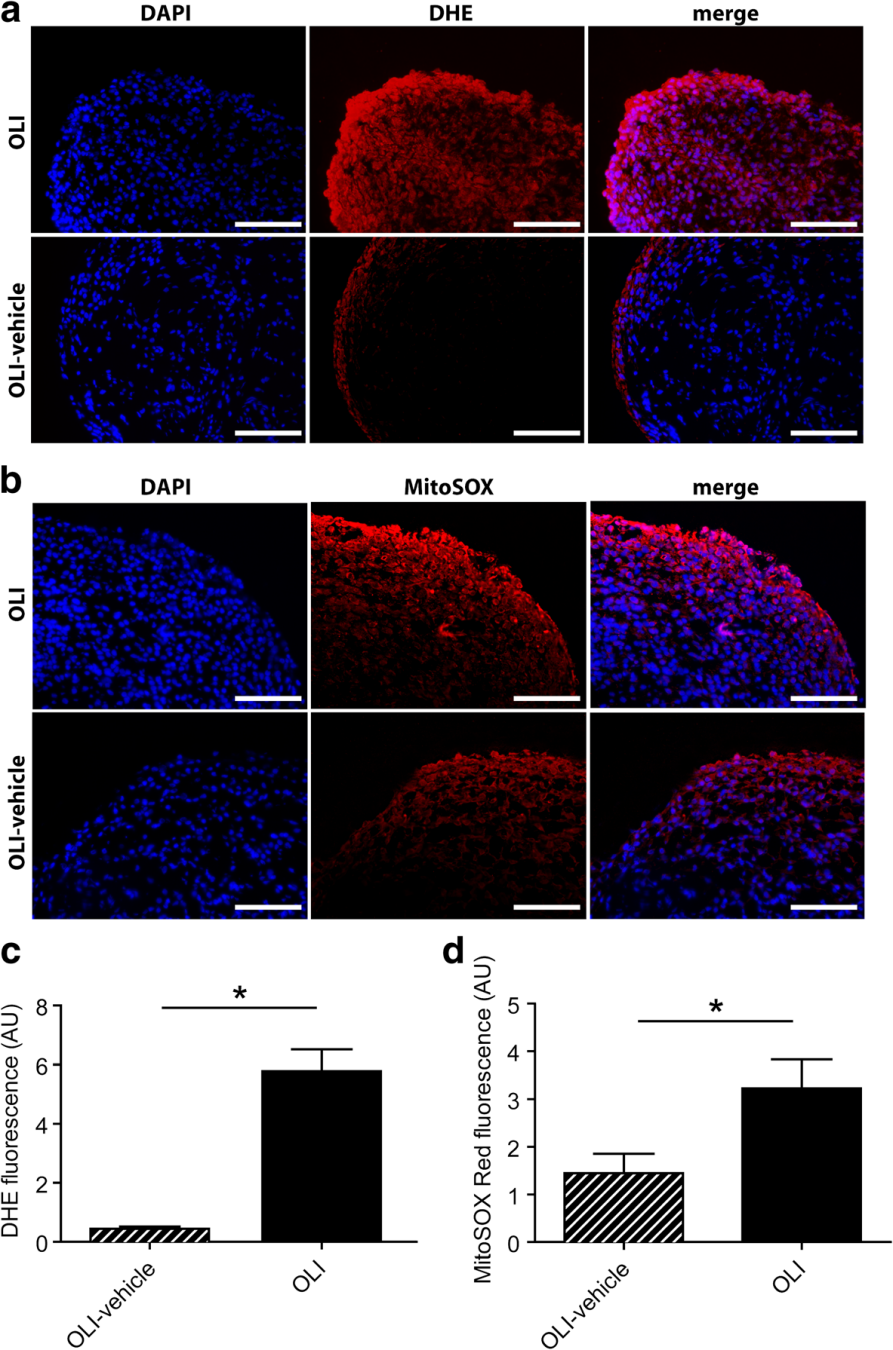
Oligomycin increases ROS production in synovial tissue. Synovial tissue frozen sections from OLI-vehicle or OLI injected joints were incubated with a cytoplasmic (DHE; **a**) or mitochondrial (MitoSox; **b**) superoxide-sensitive fluorescent dye (*red*). The nuclei were counterstained with DAPI (*blue*). Representative images of the different color channels and their merge are shown. DHE (**c**) or MitoSox (**d**) fluorescence levels were measured and calculated as described in Methods. Values, expressed in arbitrary units (AU), represent mean ± SEM (*n* = 7 independent synovial sections for each condition). *P ≤ 0.05 vs OLI-vehicle knees. OLI, oligomycin; DAPI, 4′,6-diamidino-2-phenylindole; DHE, dihydroethidium; MitoSox, MitoSOX™ *Red* Mitochondrial Superoxide Indicator

**Fig. 4:**
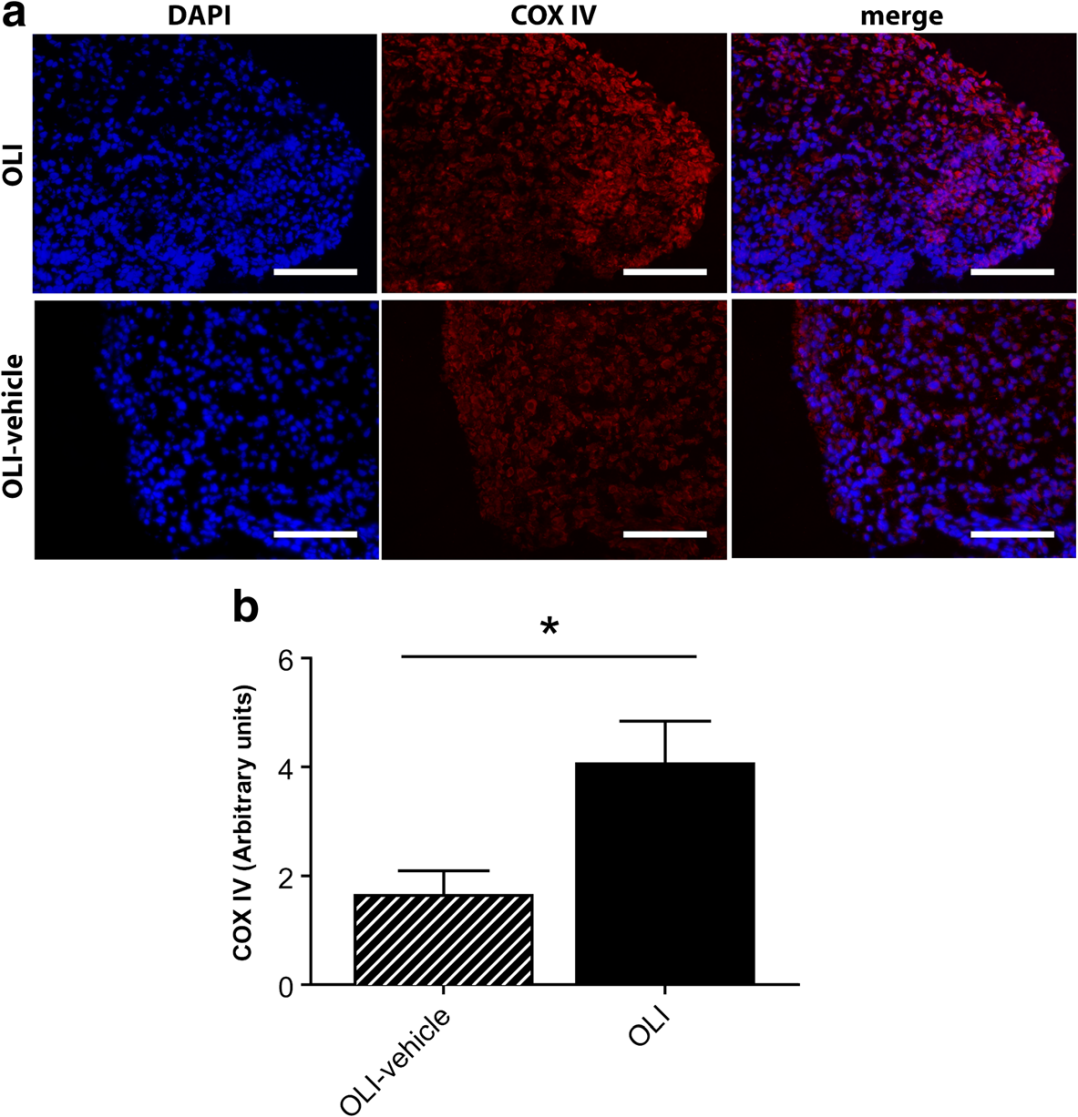
Oligomycin modulates COX-IV levels in the synovial tissue. **a** Immunofluorescence microscopy analysis of synovial tissue sections from OLI-vehicle or OLI injected joints stained for COX-IV (*red*) with DAPI counterstaining (*blue*). Representative images are presented. **b** Fluorescence levels were measured and calculated as described in Methods. Values, expressed in arbitrary units (AU), represent mean ± SEM (*n* = 7 independent synovial sections). *P ≤ 0.05 vs OLI-vehicle knees. OLI, oligomycin; DAPI, 4′,6-diamidino-2-phenylindole; COX-IV, cytochrome c oxidase subunit IV

We previously described that oligomycin induces an increase in ROS production on cultured synoviocytes and chondrocytes [30, 41]. Here, we evaluated cellular and mitochondrial ROS ex vivo in synovial tissue using superoxide-sensitive fluorophores. The results showed that synovium from oligomycin-injected joints presented a more significant increase in ROS production at both cellular (Fig. 3a and c) and mitochondrial levels (Fig. 3b and d) than from vehicle-injected control joints (*P* ≤ 0.05). We also assessed COX-IV signal since this protein is considered to be a marker of mitochondrial mass [42]. Figure 4a and b, show an increase (*P* ≤ 0.05) in COX-IV expression in the synovial tissue from the oligomycin group, as compared with the control group.

### Oligomycin exposure causes an increase in lipid peroxidation and Nrf2 activation

**Fig. 5:**
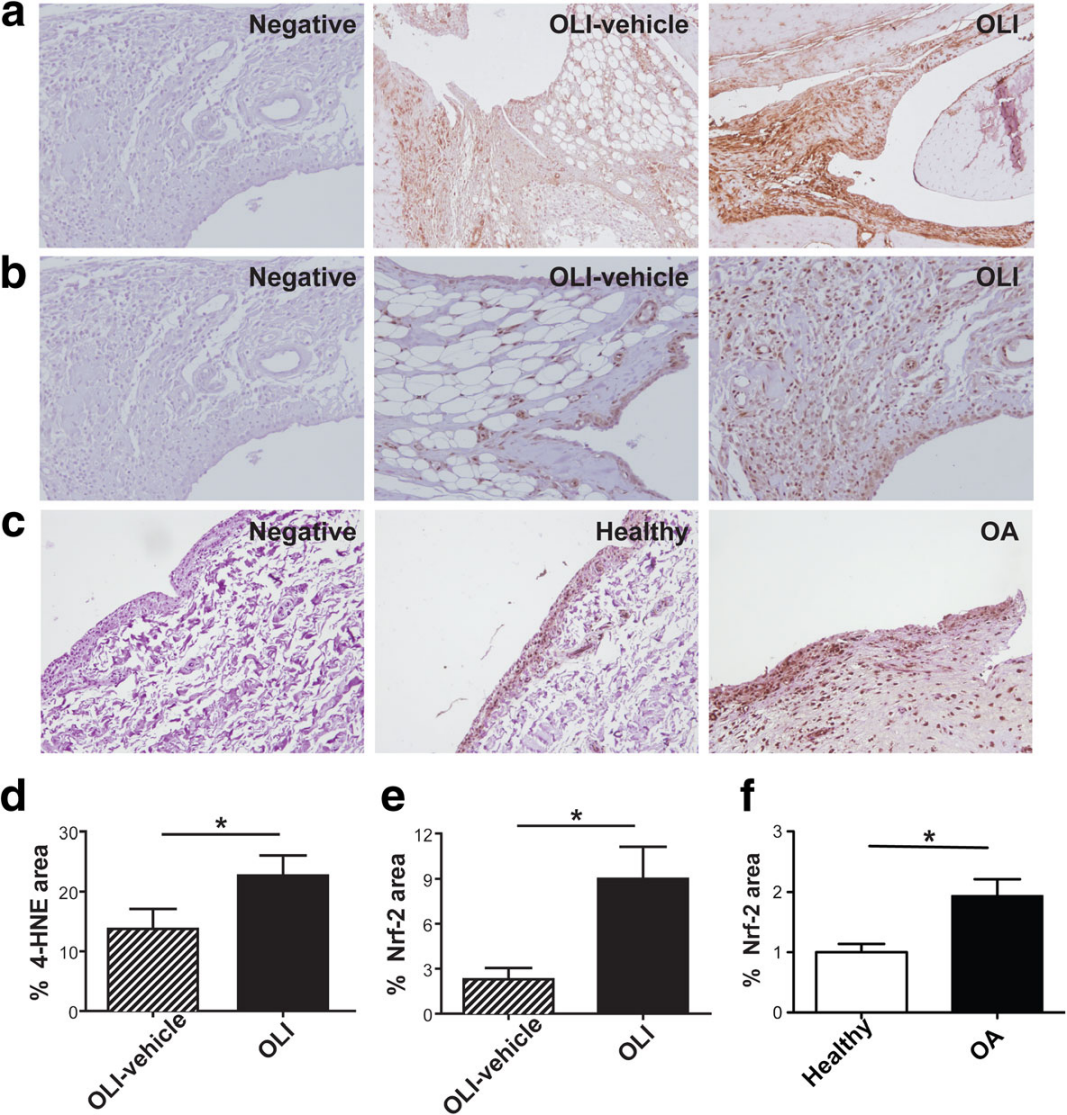
4-HNE and Nrf2 are modulated by oligomycin and Nrf2 is expressed in the synovial from OA patient. **a** Representative images of 4-HNE immunohistochemistry synovial membrane sections from OLI-vehicle and OLI injected joints. **b** Representative images of Nrf2 immunohistochemistry synovial membrane sections from OLI-vehicle and OLI injected joints. **c** Representative images of Nrf2 immunohistochemistry synovial membrane sections obtained from healthy and OA patients. A score positive immunostaining for 4-HNE (**d**) and 2 (**e**) in control and oligomycin rats is shown, and for healthy human donors and patients with OA in (**f**). Values represent mean ± SEM (*n* = 5 independent synovial tissues for rats; and *n* = 8 independent synovial tissues per each condition for human, respectively). To assess non-immune non-specific binding negative control was included in each experiment. * *P* ≤ 0.05 vs contralateral vehicle-injected knees, and vs healthy human synovium. OLI, oligomycin; 4-HNE, anti-4-hidroxi-2-nonenal; Nrf2, nuclear factor erythroid 2-related factor 2

One footprint of ROS is membrane lipid peroxidation. For this reason, we determined the presence of oxidative damage in synovial tissue by immunohistochemistry of 4-HNE, a maker of lipid oxidation. We observed a significantly greater signal of 4-HNE staining of synovial tissue from oligomycin-injected joints (*P* ≤ 0.05) than from synovial tissue from vehicle-injected control joints (Fig. 5a and d).

4-HNE is able to enhance gene expression of antioxidant and detoxifying enzymes through the redox-sensitive transcription factor Nrf2 [43]. Besides, it has been described that Nrf2 is activated in the synovial tissue of patients with RA and of arthritic mice [31]. In our model, immunohistochemical analysis of Nrf2 showed that oligomycin injection into the knee significantly increased the Nrf2 expression in the synovial tissue (*P* ≤ 0.05) (Fig. 5b and e), compared with vehicle-injected control tissue. Confirming our findings in murine joint, we observed that synovial lining from patients with advanced OA (K/L grade 3-4) presents a significantly higher Nrf2 levels compared with that from healthy patients (*P* ≤ 0.05; *n* = 8) (Fig. 5c and f).

### Oligomycin activates CINC-1 expression in the cartilage

**Fig. 6:**
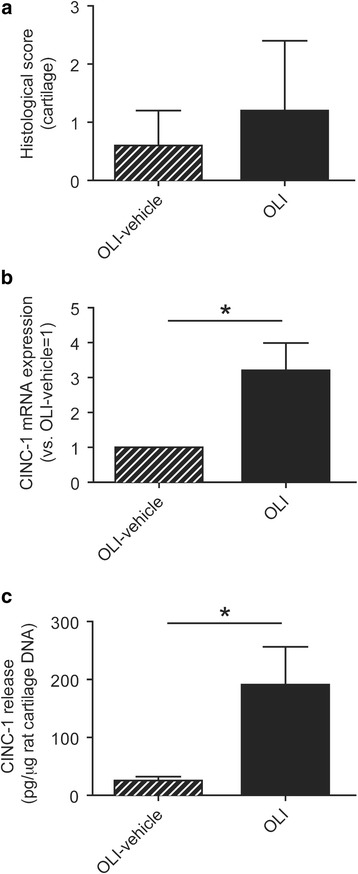
Effects of oligomycin on histopathological and inflammatory changes in the cartilage. **a** Semi-quantitative score of pathological alterations in cartilage was performed as described in Methods. Data represent mean ± SEM (*n* = 5 independent samples). **b** CINC-1 mRNA expression in cartilage. Values represent mean ± SEM (*n* = 5 samples for each condition). **c** CINC-1 protein released by cartilage explants from OLI-vehicle or OLI injected joints. Values are expressed as pg CINC-1 protein released per μg of DNA (mean ± SEM, *n* = 6 samples for each condition). *P ≤ 0.05 vs OLI-vehicle knees. OLI, oligomycin

As expected, because of the short time period of the study, no significant histopathological alterations were observed in the cartilage oligomycin-injected joints (Fig. 6a). Nevertheless, we observed modulation at molecular level in the cartilage of intra-articular oligomycin-injected joints by analyzing the pro-inflammatory chemokine CINC-1 (a murine homologue of human IL-8, commonly up-regulated in articular pathologies [5]. As shown in Fig. 6b, CINC-1 gene expression was significantly higher in cartilage from oligomycin-injected rats than from vehicle-injected control joints (*P* ≤ 0.05). To confirm at the protein level the increase in CINC-1 mRNA expression induced by oligomycin, ex vivo experiments were carried out in cartilage slices from the oligomycin group. A greater release of CINC-1 protein was observed in oligomycin-injected cartilage than in vehicle-injected control cartilage (Fig. 6c), supporting observations at the mRNA level.

## Discussion

Previous studies by other groups [11, 12, 16, 20, 22] and by us [13, 23, 25, 30, 44, 45] revealed that a decline in mitochondrial function could be implicated in arthritis pathogenesis. Most of these studies were performed under in vitro or ex vivo conditions and, strikingly, few studies have evaluated the effect of mitochondrial dysfunction on an in vivo model [17, 19, 21]. Collins et al. have shown clearly that mitochondrial DNA (mtDNA) is immunostimulatory in the mouse knee joint [19]. It has also been suggested that a decline of mitochondrial bioenergetic reserve, resulting from mitochondrial dysfunction, may contribute to a negative balance of matrix synthesis and degradation of cartilage in a spontaneous model of OA [21]. In this study, we report, for the first time, the acute effect in vivo of the intra-articular injection in the rat knee of a mitochondrial inhibitor, which can reproduce the oxidative and inflammatory status observed in arthritic joints.

The use of chemical mitochondrial inhibitors to induce mitochondrial dysfunction in in vivo models is widely accepted [46, 47]. However, the interpretation of the effect of any pharmacological intervention is always controversial because of the possibility that the drug might present off-target activities. In this sense, it is noteworthy that our model oligomycin treatment resulted in an increase of mitochondrial ROS levels, as well as an augmentation in the mitochondrial mass. The enhanced generation of mitochondrial ROS represent a key signal for mitochondrial dysfunction [10]. Besides, the increase in mitochondrial mass is a well-recognized compensatory mechanism in mitochondrial disease [48, 49].

Our macroscopic results show that oligomycin-injected joints exhibit visible articular swelling and a more significant increase of joint diameter than controls. These macroscopic determinations denoted hyperplasia and inflammatory processes in the joint that were confirmed later through histopathological and molecular analysis. Multiple evidences suggest that inflammation in synovial tissue plays an active role in the onset and progression of articular disease, such as RA [17] or OA [4, 6]. In our model, the synovial tissue in the oligomycin-injected joints presented a marked increase in damage score, showing an increase of cell layers at the lining, and a continuous infiltration of inflammatory cells at the sub-lining. In particular, a significant increase in the percentage of PMN and CD68+ cells was observed, in parallel with a significant increase in IL-8 protein expression in the synovium. Increased IL-8 expression is consistent with our previous results in vitro in synovial cells and chondrocytes in which oligomycin caused a significant increase in IL-8 mRNA and protein levels [23, 30].

A growing number of findings support the important role of mitochondrial dysfunction-elicited ROS production, and subsequently oxidative damage in chronic arthropathies [1, 3]. By using an in vitro model, we previously described that oligomycin causes both cytosolic and mitochondrial dose-dependent ROS generation in synovial cells and chondrocytes [30, 41], which is responsible of an increased IL-8 expression [23, 30]. Therefore, we hypothesized that increased oxidative stress resulting from defective mitochondria may drive the inflammatory response induced by intra-articular oligomycin exposure. When we evaluated the generation of cytosolic and mitochondrial ROS in the synovial tissue of oligomycin-injected joints, the results show that cellular and mitochondrial ROS production increase significantly more in the oligomycin-injected joints than in control joints. In relation, the increase in mitochondrial content is associated with defects in the intracellular destruction of abnormal ROS-producing mitochondria [49]. Interestingly, higher mitochondrial mass, as measured by COX-IV signal, was observed in the synovial tissue from oligomycin-injected joints than in the control joints.

Consistent with this mitochondrial failure, more oxidative damage, as measured by lipid peroxidation (4-HNE), was also observed in synovial tissue from oligomycin-injected joints. In synovial tissue from patients with inflammatory arthritis, 4-HNE is associated with a greater frequency of mtDNA mutations, which are associated with a higher presence of macroscopic features of inflammation, increased expression of pro-inflammatory cytokines, and a greater vascularization and infiltration of CD68+ and CD3+ cells [11, 12, 50]. Altogether, these data could support the idea that the inflammatory response induced in the rat knee joint by exposure to oligomycin is mediated by ROS production. To further test the relevance of oligomycin-induced oxidative and inflammatory damage, it would be interesting to investigate the effects of antioxidants.

High levels of ROS, as well as the toxic products induced by oxidative stress such as 4-HNE, are known to activate the redox-sensitive transcription factor Nrf2. For this reason, we measured by immunohistochemistry the expression of Nrf2 in the synovial tissue from oligomycin-injected joints. The results showed oligomycin significantly up-regulated Nrf2 levels. In agreement with this observation, we previously detected in vitro that exposure of synovial cells to oligomycin causes Nrf2 activation (unpublished observations), and here, a higher expression of this transcriptional factor was localized in synovial tissue from end-stage OA patients. Besides, Nrf2 activation was also observed in other articular pathologies [31]. Interestingly, it has been recently described that Nrf2 protects mitochondria against dysfunction and induces mitochondrial biogenesis [51]. Taken together, these findings suggest Nrf2 up-regulation in the joint could be a compensatory mechanism to counteract mitochondrial defects and so, it would explain higher mitochondrial mass observed in synovial tissue from oligomycin-injected joints and in OA [13].

Over-activation of the catabolic program in the chondrocyte has been suggested as an important event and a triggering mechanism that later drives the destructive processes in articular pathologies [3]. For this reason, we evaluated if mitochondrial dysfunction initially activated an inflammatory response in this tissue. Specifically, higher expression of CINC-1 (a murine homologue of human IL-8) mRNA was detected in oligomycin-injected joints. In agreement, ex vivo experiments showed increased CINC-1 protein release by cartilage explants obtained from equally treated joints. Similarly, we previously demonstrated that oligomycin increased IL-8 expression in cultured human chondrocytes [23]. CINC-1 is a chemokine that attracts neutrophils to sites where it is released. Interestingly, these inflammatory cells were localized in the synovial tissue from oligomycin-injected joints, suggesting the implication of chemokines released by the cartilage and synovium in the cellular profile of the synovial infiltrate.

## Limitations

A limitation of the current study is the use of only oligomycin for inducing mitochondrial dysfunction. Oligomycin is a specific inhibitor of mitochondrial complex V; however, a great number of findings suggest a role for impairment of other mitochondrial respiratory complexes in rheumatoid disorders [13, 17, 18, 23, 27, 30]. Thereby, in vivo studies exploring the impact of other mitochondrial inhibitors, as well as protective effects of antioxidants, should also be carried out in future. Besides, in order to evaluate the early pathological response in the joint, we performed an acute articular model of mitochondrial dysfunction. Nevertheless, evaluation of the impact of mitochondrial dysfunction at long-term evolution is recommendable in following analysis. Another concern to still be elucidated will be to investigate whether mitochondrial impairment could further aggravate the pathological pathways activated by catabolic stress in the joint, such as we have previously observed in vitro studies [23, 30].

## Conclusions

In conclusion, the present study shows, for the first time in an in vivo acute model, that oligomycin, an inhibitor of mitochondrial function, causes an oxidative and inflammatory damage in synovial joints. Our results also show that mitochondrial dysfunction in the joint increases Nrf2 expression probably as a compensatory mechanism to protect cells against oxidative and inflammatory stress generated. In summary, these data, along with our previous findings highlight the importance of mitochondrial impairment in the pathogenesis of arthropathies. Developing treatments that target the maintenance of mitochondrial homeostasis should be pursued in future studies.

## Additional file

Additional file 1: Figure S1. Representative images of complete histological sections stained with hematoxilyn and eosin**.** OLI-vehicle (**A, a**) and OLI (**B, b**) injected joints. Original magnification: A, B (4X) and a, b (20X). Higher synovial lining layer thickness (*****), as well as neovascularization (▲) and cellular infiltration (→) are observed in synovial tissue from OLI-injected joints.). M, meniscus; TP, tibial plateau; FC, femoral condyle; ST, synovial tissue. (TIFF 9220 kb)

## Abbreviations

4-HNE: 4-hydroxy-2-nonenal
BSA: Bovine serum albumin
CINC-1: Cytokine-induced neutrophil chemoattractant-1
COX-IV: Cytochrome c oxidase subunit IV
DAPI: 4′,6-diamidino-2-phenylindole
DHE: Dihydroethidium
DMSO: Dimethyl sulfoxide
HPRT: Hypoxanthine phosphoribosyltransferase
IL: Interleukin
JIA: Juvenile idiopathic arthritis
MitoSOX: mitoSOX™ red mitochondrial superoxide indicator
MRC: Mitochondrial respiratory chain
mtDNA: mitochondrial DNA
Nrf2: Nuclear factor erythroid 2-related factor 2
OA: Osteoarthritis
PBS: Phosphate buffered saline
PMN: Polymorphonuclear leukocytes
RA: Rheumatoid arthritis
ROS: Reactive oxygen species
SOD: Superoxide dismutase
