# Supplementary figures and images for: The mitochondrial inhibitor oligomycin induces an inflammatory response in the rat knee joint
Source: BMC Musculoskelet Disord. 2017 Jun 12;18:254. doi: 10.1186/s12891-017-1621-2 (PMC5469149; doi:10.1186/s12891-017-1621-2)

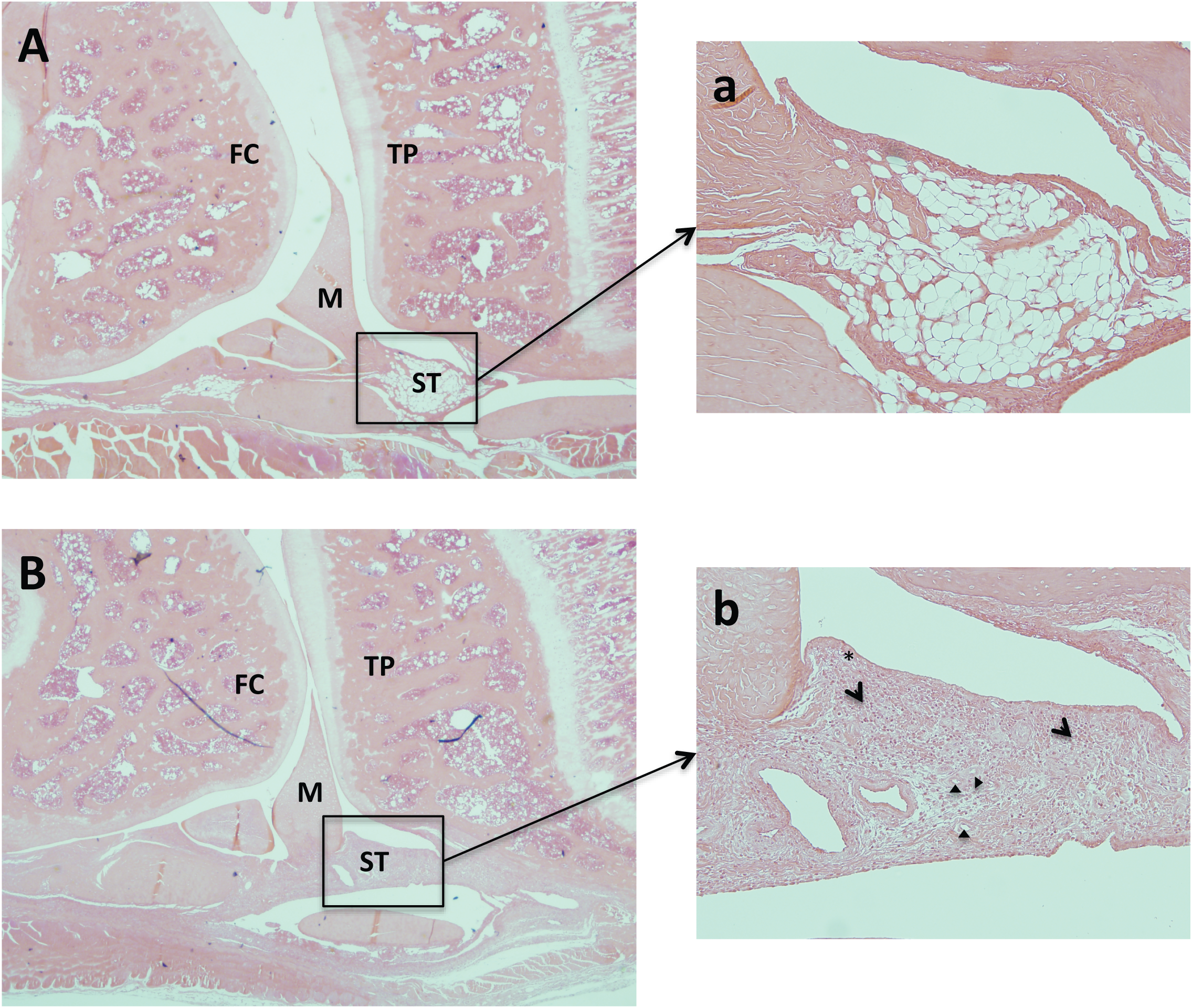

Supplement: Additional file 1: Figure S1. — Representative images of complete histological sections stained with hematoxilyn and eosin. OLI-vehicle (A, a) and OLI (B, b) injected joints. Original magnification: A, B (4X) and a, b (20X). Higher synovial lining layer thickness (*), as well as neovascularization (▲) and cellular infiltration (→) are observed in synovial tissue from OLI-injected joints.). M, meniscus; TP, tibial plateau; FC, femoral condyle; ST, synovial tissue. (TIFF 9220 kb) [file 12891_2017_1621_MOESM1_ESM.tif]
